# Cutaneous leishmaniasis in Kutaber District, Ethiopia: Prevalence, sand fly fauna and community knowledge, attitude and practices

**DOI:** 10.1016/j.heliyon.2023.e18286

**Published:** 2023-07-14

**Authors:** Abib Berhanu, Sisay Dugassa, Minwuyelet Maru, Abebe Animut, Berhanu Erko, Asrat Hailu, Araya Gebresilassie

**Affiliations:** a Addis Ababa University, College of Natural and Computational Sciences, Department of Zoological Sciences, Insect Science Stream, Addis Ababa, Ethiopia; b Aklilu Lemma Institute of Pathobiology, Addis Ababa University, Ethiopia; c Health Research and Technology Transfer Directorate, Amhara Public Health Institute, Dessie Branch, Ethiopia; d College of Health Sciences, Addis Ababa University, Ethiopia

**Keywords:** CL, KAP, Kutaber, *Phlebotomus longipes*, Ethiopia

## Abstract

**Background:**

Cutaneous leishmaniasis (CL) affects 25% of the population living in the highlands of Ethiopia. CL intervention has not decreased the number of leishmaniasis patients. A cross-sectional study was conducted to determine CL prevalence, community’s knowledge, attitude and practices (KAP), and the sand fly fauna in Kutaber district, northeast Ethiopia.

**Methods:**

A retrospective, community-based cross-sectional study was conducted in Boru Meda Hospital from December 2014–March 2021 to study CL prevalence of Kutaber district. A Pre-tested, well-structured questionnaire was used to collect data on the participants' socio-demographic characteristics, KAP towards CL and knowledge about sand fly vectors. Chi-square test and logistic regression analysis were used in the study, and data were analyzed using SPSS version 23 (p < 0.05).

**Results:**

A total of 10,002 (14.02%), of which 71,325 samples were confirmed as positive for CL. The infection rate of CL in females (7.1%) was a little bit higher than males (7.0%). More cases were recorded among 15–29 age category. The study also revealed that 77.1% of the respondents had poor knowledge about CL treatment, prevention, clinical presentation and disease transmission. Farmers tended to have poorer knowledge about sand flies than non-workers and students (32.7 vs. 35 and 44.1%; P = 0.049). Housewives had poorer knowledge about sand flies than farmers and workers (22.2 vs. 32.7 and 33.3%; P = 0.023). *Phlebotomus longipes* comprised the highest composition (80%) of the sand fly species identified in Kutaber district.

**Conclusions:**

The data showed that the community had poor knowledge about CL, vector, and transmission mode. CL preventive measures were prevalent, implying the need to raise CL awareness. *Phlebotomus longipes* was identified as the most dominant sand fly species which accounted for CL. The findings can be used in developing an effective control strategy to reduce CL transmission in the study area and elsewhere in Ethiopia.

## 1 Introduction

Leishmaniasis is a disease caused by obligate intracellular protozoan parasites in the genus *Leishmania* and transmitted by the bite of infected female phlebotomine sand fly [1]. The disease is common in 98 countries in Europe, Africa, Asia and America [2]. Leishmaniasis commonly causes three distinct clinical manifestations, namely cutaneous leishmaniasis (CL), mucocutaneous leishmaniasis (MCL) and visceral leishmaniasis (VL) [3].

Cutaneous leishmaniasis is endemic in Ethiopia and has been known since 1913 [4,5]. CL is a major cause of morbidity and disfigurement. It infects about 20,000 to 50,000 people annually [6], and over 29 million people are at risk of CL in the country [7]. The disease prevails in the highlands between 1400 and 3175 m above sea level [8,9]. The main causative agent, *Leishmania aethiopica* is transmitted by *Phlebotomus longipes* and *P. pedifer* [4,10,11], with *Procavia capensis* and *Heterohyrax brucei* serving as its reservoir host [4,12]. In addition, Hailu et al. [13] reported sporadic cases of *L. major* and *L. tropica* in humans and sand flies in few localities of the lowlands of the country [13].

The commonly reported clinical feature of CL in Ethiopia is being characterized by a single lesion and having long-lasting ulcerations after appearance [9]. However, most CL patients have crusty lesions with irregular distribution, local edema, and color changes on their skin. The lesion spreads to the mucosa simultaneously with lesions on the skin [14,15]. The lesion in CL patients has sustained for decades and eventually turned into diffused cutaneous leishmaniasis (DCL). The DCL affects wide area of the skin, with various papular, nodular, or plaque lesions that regularly lack ulceration [[16], [17], [18]].

CL is known to be endemic in the northeastern part of Ethiopia, particularly in Kutaber district surrounding Dessie town [4,[19], [20], [21]]. The epidemiology of this disease is poorly understood in most endemic areas of Ethiopia, and recent CL cases are not completely reported by health institutions such as Boru Meda Hospital (BMH) [22]. This is attributed to a lack of well-equipped health facilities and services, local climate change, and the community’s impaired immunity due to malnutrition [[22], [23], [24]]. Although CL cases have been frequently reported from health institutions in this district and the surrounding areas, there is no recent report on the knowledge, attitude and practice towards CL among rural communities of the community. There is also insufficient information about abundance of sand fly species in Kutaber district.

Studies indicated that improving community knowledge, attitude and practices (KAP) is an effective way of tackling infectious diseases because it plays an important role in the prevention and control of such diseases [25,26]. Numerous studies have been carried out in different endemic areas worldwide in order to assess KAP towards CL among various populations. On the other hand, few studies were conducted in Ethiopia to assess KAP towards CL [9,27]. Hence, the present study assesses the prevalence of disease and the risk factors of CL in Kutaber district, evaluates KAP of the community, and develops an effective control strategy in reducing the transmission of the CL in the study area and other CL endemic foci of the country.

## 2 Material and methods

### 2.1 Study area description

**Fig. 1:**
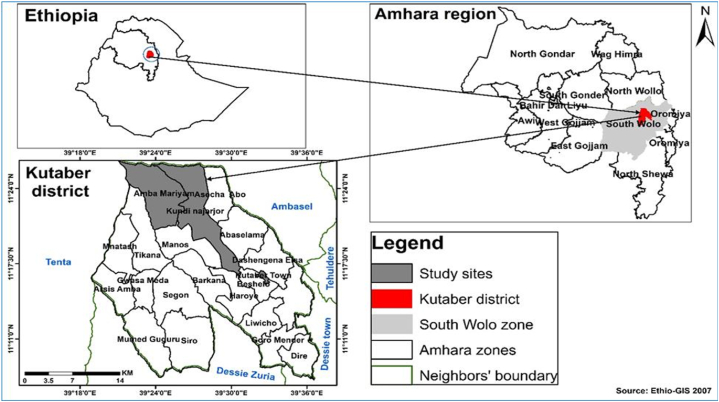
The study site of Kutaber district, South Wello Zone, Amhara Regional State, Ethiopia.

The retrospective study was carried out in Boru Meda Hospital (BMH). Three *Kebeles*, namely Kutaber town, Amba Mariyam and Kundi Najarijor were selected in Kutaber District, South Wollo Zone, Amhara National Regional State, northeast Ethiopia to study KAP and sand fly abundance (Fig. 1). Kutaber District is 422 km away from north of Addis Ababa. It is located at 11,012′36″ −11018′36″ N latitude and 39,031′12″-39034′12″ E longitude [28]. Kutaber district has highland and lowland areas with minimum and maximum rainfall ranges between 500 and 955 ml in rainy season. The annual temperature ranges from 10 to 20 °C [29]. The three aforementioned *kebeles* of Kutaber district were selected because of their high endemicity of CL among the 23 known CL-endemic areas identified by the district health office. The district covers 719.92 km^2^. It has a population of 110, 984 until May 2021, according to the census Report of the district health office. Kutaber is typically a plateau at 2650 m above sea level, having steep slopes rising to 3,000 m, and then climbing steadily to summits around 3,400 m.

To the East of the district, there lies a wide valley, which is flooded every rainy season but used as a pasture in the dry season. There is a tributary gorge leading to the Blue Nile to a depth of 2,200 m [4]. Several stone fences, termite hills, soil cracks, holes in walls, barns, caves, and rodent burrows are found in the study areas (compounds, agricultural fields, and their peri domestic areas), which are suitable for the breeding of the sand fly vectors [4,[30], [31], [32]]. BMH is one of the health institutions in the district that gives service primarily in the Dermatology and Ophthalmology Department to outpatients coming from Dessie town and the surrounding areas. It is the only center known for treating skin disease in the Amhara Region and the surrounding environments.

### 2.2 Study design, population, and data collection

#### 2.2.1 Retrospective leishmaniasis data collection

To assess the trend of CL prevalence in retrospective survey study: a 5-year CL retrospective data (2015–2019), COVID year data (from March to November 2020), and data collected until March 2021 (three-month data) were obtained from the BMH (S2). Data were recorded and collected from the patients who were positive for CL after taking microscopic, culture and Polymerase Chain Reaction (PCR) techniques. These techniques were used in the Dermatology Department of BMH to diagnose leishmania parasite in tissue specimens taken from skin wounds of different sizes, lesion number and/or types of medication.

#### 2.2.2 Sample size and sampling techniques

The WHO’s practical manual for sample size determination in health studies was used to calculate the minimum sample size required for this survey [33]. Previous data on the knowledge about and attitude towards CL in north-central Ethiopia was 69% [20]; with a 95% confidence interval and 5% level of significance and 10% contingency. Thus, 292 households were targeted and used for the probability proportional to size sampling method in order to allow for non-participation and incomplete questionnaires. Because decisions were made at the home level, the household was chosen as the sampling unit. In terms of population size, the three areas chosen are approximately identical. Hence, 97 households were selected from each Kutaber town and Amba Mariam, while 98 households from Kundi Najarjor by simple random sampling using a list compiled by health post officer in each study *kebele*.

#### 2.2.3 Measurement of knowledge and practices of participants

In order to measure KAP of the study participants about CL and its prevention and control practices, a structured questionnaire was designed and administered (S1). The questionnaire covered socio-demographic details, knowledge about CL transmission, treatment and prevention, and risk perception of the disease. It also covered bed net ownership and use, prevention and treatment practices of the respondents. In addition, participants were also asked about their knowledge on the sand fly vector. The questionnaire was first developed in English and translated into Amharic (the local language), and then pre-tested in non-selected patients for assessing content validity, appropriateness, and question comprehensibility. It was administered to 292 study participants selected from the three *kebeles* in the study area from April to May 2021. Two laboratory technicians from the health center were selected to collect data, and training was given to them on how to conduct the interview, content of the questionnaire, data quality, and ways to approach respondents. Data were checked for completeness, and incomplete questionnaires were returned to data collectors for correction by revisiting the concerned interview.

### 2.3 Species composition and relative abundance of sand flies

#### 2.3.1 Sand fly collection

For the sand fly fauna and relative abundance study, three representative *kebeles* namely Kutaber town, Amba Mariyam and Kundi Najarijor, were selected based on the occurrence of CL. Sand flies were collected from April to May 2021. Within the sampling kebeles, four representative trapping habitats, namely, indoor, peri-domestic, farm field and caves were identified and used for collecting the entire sand fly species collection. Sand flies were trapped for two nights at each sampling *kebele*, totaling 6 collection trap nights as previously done by Aklilu et al. [34] and Gebresilassie et al. [35].

Sand flies were collected using five CDC light traps (LT) and ten sticky traps (ST). Two LTs were deployed at caves; three LTs were fixed in peri-domestic habitats such as cracked walls, stone piles, and areas closer to grazing fields of animals. For the other night, three LTs were placed to sample sand flies from indoor, agricultural open fields, and areas closer to mixed forests. And the edge of farmlands. The LTS were suspended 0.4–0.5 m above the ground level. The traps were deployed an hour before sunset and collected at dawn the following morning. Then, the sand flies were sorted by sex and preserved in 70% ethanol for later species identification [35].

In addition, white sticky traps (ST) coated with sesame oil were used for capturing sand flies. Four STs were installed inside four selected houses in the study *kebeles* to capture any indoor resting (endophilic) sand flies. Similarly, another four STs were randomly installed horizontally on cracked walls, stone piles, animal enclosures in agriculture, and peri-domestic habitats. For the other night, four STs were randomly installed inside the caves. Four STs were placed horizontally on the cracks of agriculture fields while the other two sets of STs were hung vertically in a row 30 cm above the ground supported by metal pegs. Each morning, sand flies from STs were removed using forceps and they were stored in 96% ethyl alcohol in labeled vials for species identification.

#### 2.3.2 Mounting and identification of sand flies

The head (cibarium, and throat) and the last three segments of the abdomen (genitalia), which contain the spermatheca in female sand fly and the penis sheath, spines, and hair tufts in male sand fly, are the most common taxonomic traits for identifying sand flies. Two halves of each sand fly were mounted on a micro slide with Hoyer’s medium, coated with a cover slip, and left to air dry for a day or two before being identified using Abonnenc’s and Minter’s Bilingual keys [36] for the identification of sand flies of the Ethiopian Region.

#### 2.3.3 Ethical consideration

Ethical clearance was obtained from the College of Natural and Computational Science Institutional Review Board, Addis Ababa University (CNS-IRB; IRB/03/13/2021) and Amhara Public Health Institute, Dessie Branch, APHI-DB (APHI ‒ DB/R/T/T/D – 007) to conduct this research.

#### 2.3.4 Statistical analysis

The data on CL parasite prevalence in northeast Ethiopia, as well as different age groups, sexes, years, and clinical manifestations of CL, were entered into a Microsoft Excel data sheet and analyzed using SPSS version 23.0 (IBM Corp., New York, USA). The knowledge and attitude components, as well as the explanatory variables, were described using descriptive statistics such as frequency, percentage, and mean. The chi-square test was used to investigate the relationship between good knowledge scores and explanatory variables like age, gender, educational level, occupation, and household size, etc. Odd ratios (OR) were calculated with a significance level of 0.05 and a 95% of confidence interval (CI). Bivariate logistic regression analysis was used to investigate the relationship between independent and dependent variables. At a significance level of 0.05, multivariate logistic regression was performed to examine the relative contribution of each independent variable to the dependent variable. The relative abundance and fauna of sand flies were calculated for each *kebele* in Kutaber district.

## 3 Results

### 3.1 Retrospective trends of CL prevalence

**Fig. 2:**
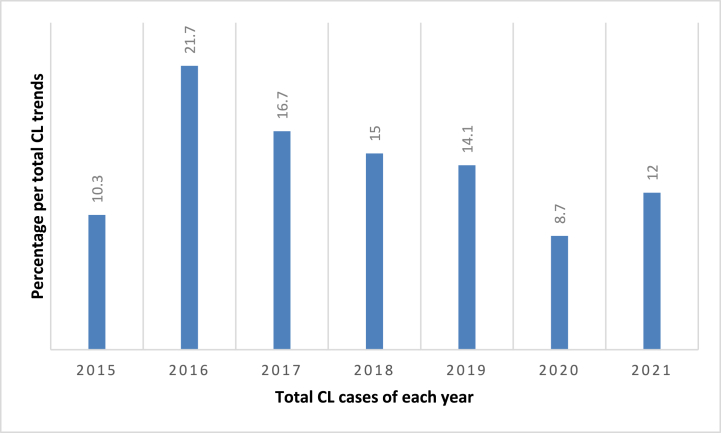
The percentage per total CL of each year trend in Boru Meda Hospital, Northeast Ethiopia, 2015–2021.

Among the five-year CL retrospective data (2015–2019), 9-month data (from March to November 2020) during COVID 19 outbreak, and a three-month data during the study period (until March 2021), a total of 71,325 CL suspected patients were diagnosed at BMH (Table 1). Out of these, 10,002 (14.02%) were found positive for the parasite. The number of CL treated cases progressively increased from 2015 to 2019 (Table 1). The highest prevalence of CL cases was observed in the year 2016 where 1718 (21.7%) patients were positive among 7911 visitors. A comparative but lower prevalence was also observed during the year 2017 with rate of 16.7% (1783 cases out of 10,676 visitors) (Fig. 2). However, in 2020, BMH was not functional due to COVID-19 pandemic and thus the data was not complete. Yet, CL cases raised up again in 2021.

**Table 1:** The prevalence of CL among patients who visited the Boru Meda Hospital (BMH) Dermatology Department from 2015 to March 2021.

Year	Examined			Positive cases		
	Male, n (%)	Female, n (%)	Total	Male, n (%)	Female, n (%)	Total
2015	3344 (49.4)	3432 (50.6)	6776	380 (5.6)	320 (4.7)	700 (10.3)
2016	4010 (50.7)	3901 (49.3)	7911	790 (10.0)	928 (11.7)	1718 (21.7)
2017	5473 (51.3)	5203 (48.7)	10,676	840 (7.9)	943 (8.8)	1783 (16.7)
2018	6200 (49.1)	6432 (50.9)	12,632	920 (7.3)	980 (7.7)	1900 (15.0)
2019	7768 (48.2)	8332 (51.8)	16,100	1180 (7.3)	1090 (6.8)	2270 (14.1)
2020**	6106 (46.6)	7002 (53.4)	13,108	590 (4.5)	545 (4.2)	1135 (8.7)
2021*	2059 (50.0)	2063 (50.0)	4122	264 (6.4)	232 (5.6)	496 (12.0)
Total	34,960 (49.0)	36,365 (51.0)	**71,325**	4964 (7.0)	5038 (7.1)	**10,002** (14.02)

CL, cutaneous leishmaniasis, n, number, %, percent, ** COVID year data, * data was collected only up to March 2021.

### 3.2 CL cases by age and sex

**Fig. 3:**
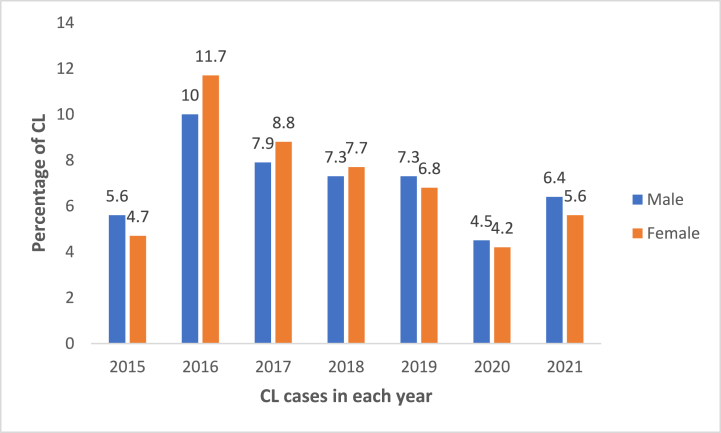
The percentage of CL cases among sex groups in Boru Meda Hospital, Northeast Ethiopia, 2015–2021.

In both sex groups, the highest prevalence of CL was seen in the year 2016 and the lowest in 2020. The trend showed regularity in the decrement of the CL cases in both sex groups from 2016 towards 2020. Females had a little bit higher CL case prevalence than males from 2016 to 2018 (Fig. 3).

**Fig. 4:**
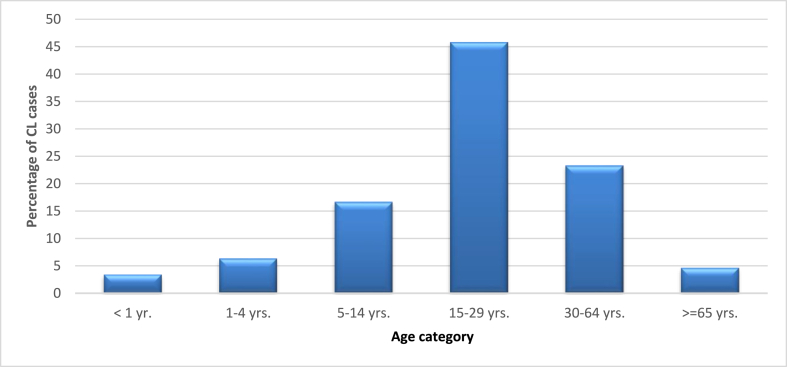
The percentage of CL cases among different age groups in Boru Meda Hospital, northeast Ethiopia, (2015–2021).

Among the age groups, 15–29 years category showed the highest CL prevalence in the year 2018. In contrast, children younger than 1 year and adults older than 65 years had low CL cases. There was no CL report observed in the year from 2018 to 2021. The next highest trend was recorded in the age group 30–64 years, followed by the age group 5–14 years. Age specific prevalence of CL is shown in Fig. 4. There was a statistically significant association between CL burden and age groups (χ2 = 3179.4, d.f. = 5, P < 0.001). The Age group 15–29 years were affected the most, with a prevalence rate of 4579 (45.8%), followed by the Age groups 30–64 years old and 5–14 years old, with prevalence rates of 2332 (23.3%) and 1666 (16.7%), respectively.

### 3.3 Clinical forms of cutaneous leishmaniasis

Among 10,002 leishmaniasis positive cases, 4526 (45.2%) were localized cutaneous leishmaniasis (LCL); 3087 (30.8%) were diffuse cutaneous leishmaniasis (DCL); and 2389 (23.8%) were mucocutaneous leishmaniasis (MCL) cases. The data showed that the majority of the cases were localized, particularly at the nose and cheek. Most of the cases (over 50%) were referred from Kutaber, followed by Delanta, Haik, and Dessie Zuria. A few cases were referred from all other parts of the country. The duration of lesions, treatment, and treatment outcomes was not available in the patient medical records. Similarly, data for the four different diagnostics clinical, skin slit smear, fine-needle aspiration cytology, and culture) that are reported and practiced in the hospital were not available in the log book.

### 3.4 Knowledge, attitude and practices (KAP)

#### 3.4.1 Socio-demographic characteristics of KAP subjects

Of the 292 study participants, 169 (58%) were males and 123 (42%) were females. Most of the participants (90, 31%) were aged between 25 and 33 years, where the median age was 29. Regarding education, 23% of the participants were illiterate. Most of the participants (190, 65%) in this survey were married, of which 92% of them lived in these localities more than five years. Around 57% of the participants were farmers. Most of the participants, 264 (90.4%) in this survey did not travel from their home in the past six months.

#### 3.4.2 Knowledge about cutaneous leishmaniasis, sand fly vector, and attitude towards CL

The first row of Table 2 shows the participants' knowledge of the signs and symptoms, transmission, prevention, and treatment of CL. In this study, 85.6% (250/292) of the participants encountered a CL case previously. When asked about the signs and symptoms of CL, 28.8% (84/292) correctly identified a lesion as the primary symptom, while 13.4% were not able to name any signs or symptoms of CL, and 21.6% indicated skin scar as the second sign and symptoms of CL. Only 28.8% (84/292) realized that CL is transmitted by sand flies, whereas 23.3% (68/292) could not name any mode of CL transmission. Furthermore, 48% of the participants had misconceptions about CL transmission by stating, for instance, mosquitoes and other flies, autoinfection, and direct person-to-person skin contact as means of CL transmission. Table 2 also shows that 34.9% of the participants correctly identified summer as the peak season for CL, whereas 25.7% were unaware of the main transmission season for CL. Regarding participants' sources of information, the majority (37.6%, 110/292) learned about the disease through family, relatives, and friends, while 26% (76/292) had personal experience of the infection. Another 9.9% (29/292) and 10.3% (30/292) learned about CL from educational institutions and social media, respectively.

**Table 2:** Knowledge, attitude towards CL, and Sand fly vectors among the study participants (n = 292).

Knowledge Types	Variables	Response categories	n (%)
Knowledge about CL	Have seen individuals infected with CL	Yes	250 (85.6)
		No	40 (13.7)
		I don’t know	2 (0.7)
	Signs and symptoms of CL	Skin wound	33 (11.3)
		Lesion	84 (28.8)
		Emaciation skin scar	31 (10.6) 63 (21.6)
		Itching and redness	13 (4.5)
		Skin ulcer	29 (9.9)
		I don’t know	39 (13.4)
	Mode of transmission of CL	Sand fly biting	84 (28.8)
		Bodily contact with patients	56 (19.2)
		Autoinfection	28 (9.6)
		By other flies	56 (19.2)
		I don’t know	68 (23.3)
	The peak incidence of CL	Summer	102 (34.9)
		Winter	46 (15.8)
		Autumn	25 (8.6)
		Spring	44 (15.1)
		I don’t know	75 (25.7)
	CL treatment options	Cauterizing	48 (16.4)
		Chemotherapy	68 (23.3)
		Herbal medicine	110 37.7)
		Religious solution	61 (20.9)
		I don’t know	5 (1.7)
	CL preventive measures	Vector control Insecticide treated net Treating patients Traditional medication Isolating patients Improving awareness I don’t know	40 (13.7) 17 (5.8) 45 (15.4) 42 (14.4) 24 (8.2) 67 (22.9) 57 (19.5)
Attitude towards CL	Is CL more dangerous than malaria?	Yes	123 (42.1)
		No	82 (28.1)
		I don’t know	87 (29.8)
	Is CL a curable disease?	Yes	134 45.9)
		No	88 (30.1)
		I don’t know	70 (24)
	Is CL a preventable disease?	Yes No I don’t know	79 (27.1) 178 (61) 35 (12)
Knowledge about sand fly vectors	Can you identify sand flies from other flies and mosquitoes?	Yes No	22 (7.5) 270 (92.5)
	Causes of CL transmission	Bite of sand fly	84 (28.8)
		Bodily contact with patients	56 (19.2)
		Autoinfection	28 (9.6)
		By other flies I don’t know	56 (19.2) 68 (23.3)
	Places in which sand fly commonly found	Everywhere	4 (1.4)
		Cracks in basalt cliffs	68 (23.3)
		Fissures	12 (4.1)
		Caves used by hyrax	84 (28.8)
		Termite nests	11 (3.8)
		Rodent burrows	11 (3.8)
		Soil cracks	12 (4.1)
		Tree trunks	4 (1.4)
		I don’t know	86 (29.5)
	Biting time of sand flies	Night	128 (43.8)
		Any time	29 (9.9)
		Morning	17 (5.8)
		Day time	22 (7.5)
		From dusk till dawn	18 (6.2)
		I don’t know	78 (26.7)
	Methods to control sand flies	Uses of insecticides on	
		animal shelter	48 (16.4)
		Space-spraying	37 (12.7)
		Insecticides treated nets	77 (26.4)
		Personal hygiene	48 (16.4)
		Personal protection	56 (19.2)
		I don’t know	26 (8.9)

The results of the participants' attitudes toward CL are shown in the second row of Table 2. CL was considered a serious condition by 123 (42.1%) people, who believed that it was more harmful than malaria, whereas 28.1% people took it as a mild infection. Surprisingly, many of the participants (45.9%) had a positive attitude and believed the disease could be cured, while only 30.1% people believed that the disease could not be cured. However, 61% of those interviewed said that CL could not be prevented. It is clear from the analysis of these results that more than 80% of the participants had a negative attitude regarding CL (Table 2).

The participants' knowledge about sand flies and their role in disease transmission is summarized in the third row of Table 2. Surprisingly, nearly all of the participants (92.5%) were unable to recognize and distinguish sand flies from other flies. Nevertheless, 28.8% (84/292) of the participants mentioned sand flies as a source of CL transmission. Most of the participants (23.3%) had no perception of whether sand flies might transfer diseases or not. Similarly, 19.2% of the participants believed that other flies may transmit CL. Caves used by hyrax and cracks in basalt cliffs were mentioned as breeding places by 28.8% (84/292) and 23.3% (68/292) of the participants, respectively; while the majority (29.5%) did not know about such breeding places. In terms of biting time, 43.8% of participants accurately indicated that sand flies bite during night time, whereas 9.9% stated that sand flies bite at any time of day. Around 9.0% of the participants were unable to mention any methods that control sand flies, while 35.9% responded that sand flies can be controlled using personal hygiene and good personal protection. The aforementioned findings revealed that nearly two-thirds of the respondents (65.1%) had poor knowledge of the sand fly as a CL vector.

#### 3.4.3 Practices of study participants towards CL prevention and control

In terms of treating the disease, 37.7%, 23.3%, and 16.4% of the study participants mentioned herbal medication, chemotherapy, and cauterization as methods of treatment for CL, respectively. Only 22.9%, 13.7% and 15.4% of them identified ‘improving awareness’, ‘controlling the vector’ and ‘treating infected patients’ as preventive measures, respectively. This agrees with the low usage of Insecticide-treated nets (ITNs) (5.8%). Furthermore, almost one-fifth (19.5%) of the study participants do not know how to prevent CL in their residential areas. Based on the aforementioned findings, the majority of the participants (77.1%) had poor knowledge about CL (Table 3).

**Table 3:** Scores of knowledges and attitude towards cutaneous leishmaniasis and sand fly vectors among the participants (n = 292).

Characteristics (total score options)	Knowledge and attitude scores (interpretation)	n	%
Knowledge towards CL (5)	0-3 (poor) 4-5 (good)	225 67	77.1 22.9
Knowledge towards Sand fly (5)	0-3 (poor) 4-5 (good)	190 102	65.1 34.9
Attitude about CL (3)	0-1 (Negative attitude) 2-3 (Positive attitude)	239 53	81.2 18.2

#### 3.4.4 Factors associated with knowledge about and attitude towards CL and the sand fly vectors

Table 4 shows the association of participants' knowledge towards CL and sand fly vector with their socio-demographic factors. Significantly higher proportion of male participants had better CL knowledge than females (29% vs. 15%; P = 0.005). The percentage of participants with good knowledge of sand flies was lower among farmers than among the non-workers and students (32.7 vs. 35 and 44.1%; P = 0.049). Similarly, the percentage of participants with good knowledge of sand flies is significantly lower among housewives than farmers and workers (22.2 vs. 32.7 and 33.3%; P = 0.023). The distribution of scores for a positive attitude toward CL is comparable among all age groups except 25–33 years (P > 0.05).

**Table 4:** Association of participants' knowledge towards cutaneous leishmaniasis and Sand fly vector with their socio-demographic factors (n = 292).

Variables	Good knowledge about CL^k^			Good knowledge about Sand fly^k^		
	n (%)	COR (95% CI)	AOR (95% CI)	n (%)	COR (95% CI)	AOR (95% CI)
Age (years)						
16–24	8 (12.5)	0.54 (0.177–1.638)	0.16 (0.020–1.248)	29 (45.3)	0.64 (0.327–1.240)	0.35 (0.083–1.500)
25–33	44 (68.8)	0.08 (0.032–0.203)*	0.02 (0.003–0.100)*	27 (30)	1.23 (0.651–2.326)	1.24 (0.439–3.531)
34–41	9 (14.1)	0.39 (0.129–1.151)	0.19 (0.048–0.783)*	17 (31.5)	1.15 (0.553–2.380)	0.91 (0.351–2.367)
≥42	6 (9.4)	1	1	29 (34.5)	1	1
Gender						
Male	49 (29)	0.42 (0.230–0.765)*	0.29 (0.091–0.894)*	71 (42)	0.47 (0.280–0.774)*	0.36 (0.168–0.792)*
Female	18 (15)	1	1	31 (25.2)	1	1
Education						
Unable to write and read	11 (16.4)	2.91 (0.726–11.658)	4.56 (0.157–132.154)	17 (25.3)	1.10 (0.262–4.639)	0.40 (0.021–7.737)
Primary	5 (15.2)	3.20 (0.677–15.136)	21.90 (0.694–690.638)	13 (39.4)	0.58 (0.129–2.584)	0.24 (0.012–4.630)
Secondary	10 (18.5)	2.51 (0.615–10.271)	9.83 (0.424–227.545)	23 (42.6)	0.51 (0.121–2.117)	0.41 (0.027–6.343)
Tertiary	6 (31.6)	1.24 (0.259–5.913)	–	6 (31.6)	0.81 (0.157–4.197)	–
No formal education	13 (26.5)	1.58 (0.397–6.306)	2.45 (0.093–64.734)	17 (34.7)	0.71 (0.165–3.014)	0.34 (0.018–6.456)
Pre-secondary	18 (30.5)	1.30 (0.338–5.009)	2.01 (0.088–45.730)	23 (39)	0.59 (0.141–2.444)	0.33 (0.019–5.692)
Preparatory	4 (36.4)	1	1	3 (27.3)	1	1
Religion						
Orthodox Christian	23 (20)	4.00 (0.241–66.388)	–	36 (31.3)	–	–
Muslim	43 (24.6)	3.07 (0.188–50.134)	–	66 (37.7)	–	–
Others	1 (50)	1	–	0 (0)	–	–
Occupation						
Student	11 (32.4)	0.47 (0.127–1.705)	0.04 (0.001–1.193)	15 (44.1)	1.52 (0.517–4.468)	1.97 (0.216–18.020)
Private employee	0 (0.00)	–	–	2 (33.3)	2.40 (0.361–15.942)	1.52 (0.044–52.872)
Housewives	9 (33.3)	0.44 (0.116–1.709)	1.69 (0.074–38.661)	6 (22.2)	4.20 (1.220–14.454)*	2.50 (0.233–26.746)
Farmer	30 (18.2)	1.00 (0.316–3.169)	1.03 (0.075–14.252)	54 (32.7)	2.47 (1.003–6.067)*	4.10 (0.628–26.798)
Not working	7 (35)	0.41 (1.00–1.708)	0.11 (0.003–4.379)	7 (35)	2.23 (0.642–7.735)	0.78 (0.045–13.680)
Government employee	6 (33.3)	0.44 (0.103–1.915)	–	6 (33.3)	2.40 (0.661–8.720)	–
Marchant	4 (18.2)	1	1	12 (54.5)	1	1
Household members						
2	6 (23.1)	0.83 (0.297–2.334)	11.06 (0.890–137.439)	6 (23.1)	1.81 (0.670–4.912)	1.31 (0.238–7.216)
3–5	40 (24.8)	0.76 (0.416–1.374)	1.00 (0.346–2.924)	59 (36.6)	0.94 (0.563–1.571)	0.96 (0.462–1.989)
>5	21 (20)	1	1	37 (35.2)	1	1
Marital status						
Married	47 (24.7)	0.85 (0.297–2.401)	4.60 (0.741–28.532)	62 (32.6)	1.33 (0.545–3.234)	1.97 (0.549–7.076)
Unmarried	14 (19.4)	1.15 (0.364–3.634)	134.51 (7.350–2461.797)*	29 (40.2)	0.95 (0.365–2.491)	4.08 (0.623–26.735)
Divorced	1 (14.3)	1.67 (0.161–17.257)	15.13 (0.619–369.677)	2 (28.5)	1.61 (0.255–10.132)	1.05 (0.117–9.518)
Widowed	5 (21.7)	1	1	9 (39.1)	1	
Possession farm land						
Yes	42 (20.8)	1.47 (0.826–2.598)	–	71 (35.1)	0.97 (0.575–1.634)	–
No	25 (27.8)	1	–	31 (34.4)	1	–
Distance of farmland from home						
<100 mt.	8 (15.4)	1.67 (0.640–4.380)	–	19 (36.5)	0.69 (0.310–1.522)	0.57 (0.229–1.439)
100–1000 mt.	20 (22.2)	1.08 (0.497–2.350)	–	36 (40)	0.60 (0.300–1.218)	0.44 (0.185–1.032)
>1000 mt.	14 (23.3)	1	–	17 (28.3)	1	1
Duration of living in the province						
1–2 yrs.	2 (13.3)	2.03 (0.446–9.231)	–	5 (33.3)	1.04 (0.345–3.131)	2.79 (0.280–27.872)
3–5 yrs.	1 (12.5)	2.19 (0.264–18.098)	0.75 (0.027–21.064)	5 (62.5)	0.31 (0.073–1.334)	0.30 (0.044–1.972)
>5 yrs.	64 (23.8)	1	1	92 (34.2)	1	1
Have you travelled out in the last six months?						
Yes	1 (3.6)	9.00 (1.200–67.525)*	–	13 (46.4)	0.59 (0.268–1.287)	0.58 (0.179–1.853)
No	66 (25)	1	–	89 (33.7)	1	1

All values are numbers (%). COR, Crude Odds Ratio; AOR, Adjusted Odds Ratio, CI, Confidence Interval.

*Significant association (P < 0.05).

^k^Based on scores shown in Table 3.

**Fig. 5:**
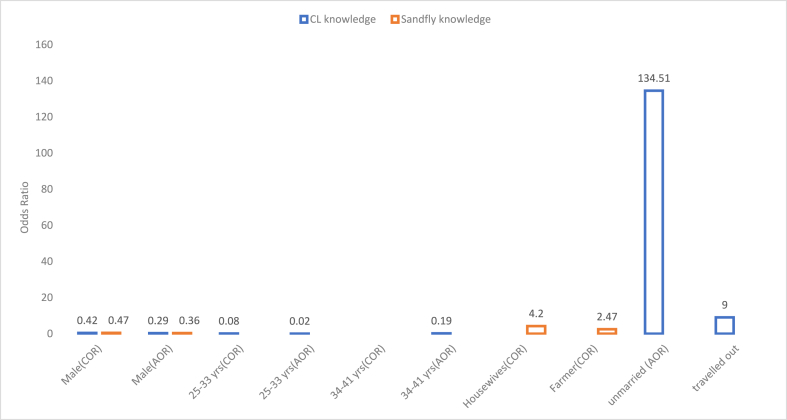
Predictors for knowledge towards cutaneous leishmaniasis and Sand fly. COR, Crude Odds Ratio; AOR, Adjusted Odds Ratio.

Fig. 5 illustrates age and gender as significant predictors of good knowledge for CL and sand flies, respectively. Adults in the age group of 25–33 years old were 20% times more likely to have good knowledge of CL, and 36% males more likely to have good knowledge of sand fly. Occupation was described as the significant factor of good knowledge towards sand fly based on Bivariate logistic regression. Hence, housewives were 1.70 times more likely to have a better knowledge of sand flies than farmers (COR = 2.47).

In order to clarify the results presented in Table 4, the variables which showed significant difference were summarized and presented in Fig. 5.

#### 3.4.5 Species composition and relative abundance of sand flies

Table 5 shows the relative abundance and fauna of sand flies from Kutaber district. A total of 105 sand fly specimens comprising 5 species in two genera (*Phlebotomus* and *Sergentomyia*) were collected using LTs and STs during the collection nights. Overall, 2 and 3 sand fly species in the genera *Phlebotomus* and *Sergentomyia* were identified, respectively. Thirty eight, 23 and 44 specimens of sand fly were identified in *kebeles* of Kutaber town, Kundi Najarjor and Amba Gibi of Kutaber district, respectively. In Kutaber town, three species were caught, comprising *Phelbotomus longipes*, *Sergentomyia bedfordi*, and *S. africana*. *Phelbotomus longipes* comprised the highest composition (80%) of all the described species; while *P. sergenti* and *S. squamipleuris* made up the lowest number of species.

**Table 5:** Relative abundance and fauna of sand flies collected from the Kutaber district, April to May 2021.

Sand fly species	Types of collection methods								Overall total Kut. Kun. A/G Tot.				Relative frequency (%)
	Light traps Sticky traps												
	Kut. Kun. A/G Tot. Kut. Kun. A/G Tot.												
*Phlebotomus longipes*	16	13	20	49	19	2	14	35	35	15	34	84	80
*Phlebotomus sergenti*	0	1	1	2	0	0	0	0	0	1	1	2	1.9
*Sergentomyia squamipleuris*	0	0	1	1	0	0	2	2	0	0	3	3	2.9
*Sergentomyia bedfordi*	0	4	3	7	1	0	2	3	1	4	5	10	9.5
*Sergentomyia africana*	0	2	0	2	2	1	1	4	2	3	1	6	5.7
Total	16	20	25	61	22	3	19	44	38	23	44	105	100.0

*Note:* Kut, Kutaber; Kun, Kundi; A/G, Amba Gibi; Tot, Total.

## 4 Discussion

The Federal Ministry of Health has been using IRS and LLITNs to control malaria and other tropical disease vectors in endemic areas of the country since 2006. This study examined CL data of the previous five years, data from the COVID 19 outbreak, and the records until March 2021 of BMH’s Dermatology Department. The aim of this study was to assess CL trends in the BMH, identify sand fly species, and investigate factors that can influence CL knowledge, attitudes, and practices in the Kutaber district of northeast Ethiopia.

In all years of retrospective study in BMH, the overall prevalence of CL was 14.02%, revealing that the localities in the catchment of the health facility are important CL foci in northeast of the country. The overall positivity rate of CL in the present study is lower than the prevalence reported by similar health facility-based studies in Ethiopia and elsewhere [[37], [38], [39]]. This may be due to the different study periods, disease reawakening, the various socio-economic status of society, the scarcity of health facilities, and the knowledge and attitude of the community towards the CL [27]. This study showed the presence of a localized form of CL, which is in agreement with similar studies in other parts of Ethiopia [38,40,41].

CL prevalence in the BMH is higher in the age group 15–29 years, possibly due to the socioeconomic activity of the residents in the study area. Its transmission may have occurred in peri-domestic habitats, where sand fly exposure is more evenly distributed among individuals. This is evidenced by the fact that the disease was almost equally distributed between both sexes in the study, with large numbers of women and men, including adults and the elderly, infected. This could be due to the active participation in agricultural activities of matured family members, who are highly exposed to sand fly in the northeastern part of Ethiopia. All age groups are susceptible, according to Lemma et al. [42]. However, most cases occur in groups that have regular contact with sand fly habitats. Similar findings indicated that the number of patients admitted for cutaneous leishmaniasis was higher than that of the 16-year-old patients [20,21].

The present study in the BMH revealed that the localities in northeastern Ethiopia are important CL foci of the country. The clinical manifestations of most cases were of the Localized Cutaneous Leishmaniasis (LCL) type throughout the years from 2015 to 2021. This result is in line with Abdela et al., van Griensven et al., and Yohannes et al. [38,40,41]. They analyzed that LCL is the most frequent manifestation followed by Mucocutaneous Leishmaniasis, and Diffused Cutaneous Leishmaniasis. This might be due to less responsiveness to treatment, disfigurement, chronic, progressive course, and non-responsiveness to the common antileishmanial drugs in MCL and DCL, respectively in Ethiopia [40]. Since CL is mostly found in malaria-free highland locations, integration with malaria management via ITN distribution and indoor residual spraying looks uncertain. Communities in this area are not aware of the rational use of insecticide and application of the malaria control strategies for Sand fly vectors [43].

The current study also evaluated CL related KAP of people in a highly endemic area of CL in Kutaber district, northeast Ethiopia. Several findings reviewed that Cutaneous leishmaniasis is well-known in the targeted population. Seife et al. [44] reviewed that CL has various vernacular names in different parts of Ethiopia, including *bolbo* in Ocholo, *finchottu* in Central Shoa, *shahegne* in North Shoa, *kunchir* in Gojam, Gondar, and portions of Wollo, *giziwa* in Tigray, *chewie* in Sodo, and *simbirahalkm* in Wollega. The shape of the lesion, the aesthetic and social stigmata associated with an illness, and the disease history are all factors that influence local vernacular names [45,46]. A skin lesion on the face (facial lesions) was considered a major symptom of CL by the participants in the current study.

Most of the participants (85.6%) observed CL cases in the community, either among family members or other people in the neighborhood. This appears to be a direct effect of the high endemicity of CL in the study areas, which causes the public to be aware of the disease signs and symptoms. Unfortunately, a comparison of the current findings with earlier findings on the amount of knowledge regarding CL and/or CL-related stigma among the Kutaber district population is impossible due to lack of previous investigations. However, this finding is in consistent with a study conducted in Ochello, southern Ethiopia (an endemic area), in which 67.6% of participants said they had encountered CL cases. Skin lesions were found to be the most common symptom with 62.2% [47]. Additionally, 77.2% CL cases and 47.1% skin lesions were found in Gondar, Northwest Ethiopia [27]. On the contrary, in the knowledge, attitude, and practice (KAP) survey conducted in Alexandria, Egypt, most participants (90%) informed that they had never seen an infected person [48].

Although most of the participants in the current study were aware of CL symptoms, they were surprised to learn that the sand fly is the disease vector. Only 7.5% of the participants identified and distinguished the sand fly as the disease vector. These findings are nearly comparable with the previous study conducted in Northwest Ethiopia and Saudi Arabia's endemic areas [27,49]. The current findings also suggested that a significant number of participants (61.6%) had misconceptions about the mechanism of transmission, with some having a belief that body (physical) contact with patients and autoinfection might be probable causes of CL. This finding is in consistent with research conducted in Hail Region, Ochello, and Gondar; in which the majority of participants had misconceptions about CL transmission in Saudi Arabia, southern and northwest Ethiopia, respectively [27,47,50]. On the other hand, studies conducted in Nepal, Brazil, and Iran found higher levels of knowledge of CL transmission [25,[51], [52], [53]]. This disparity in knowledge levels among countries may be due to misconception of social beliefs and cultural factors in CL transmission. However, it is also crucial to note that the Leishmania transmission cycle has distinct characteristics that vary from one endemic area to the next depending on the geoclimatic parameters of the study setting. Consequently, taking and concluding the results of one area and made conclusion to another is not advised.

In terms of attitudes concerning CL, most of the participants (42.1%) in this survey considered it as a more severe disease, more harmful than malaria. The high endemicity of CL in the targeted area, as well as the chronicity of the accompanying lesions that result in disfiguring scars, may explain this attitude. Such scars cause severe psychological and social problems, such as stigma, social rejection, and mental distress [54]. In general, more than 80% of the participants in this study had negative attitude towards CL. This could be a direct result of a lack of access to CL-related information. Unfortunately, a negative attitude can lead to a delay in obtaining treatment, which bring long-lasting ulceration after appearance, crusty lesions with irregular distribution, local edema, and color changes. This lesion on the skin finally spread to the mucosa present simultaneously with lesions on the skin changed to diffused form [9,14,15] and brought severe psychosocial effects in the study provinces of the Kutaber district.

The vast majority of those who took part in this study were unable to distinguish between sand flies and other flies. The difficulty of the participants to recognize this dipteran may be attributed to the fact that many members of participants do not know the causes of CL correctly (misconception that CL is caused by urine of bats). Their domestic animals dwell under the same roof; there were no separate rooms for animals in all of study areas due to the threat of wild animals around their dwelling areas. Even though few participants could identify and differentiate sand flies from other flies, they were unaware of the phlebotomine sand fly’s role in CL transmission.

Furthermore, most of the participants were unfamiliar with the locations of sand fly breeding areas and some control approaches. In contrast, a previous study in Isfahan, Iran, found that while 89.8% of participants were aware of the Sand fly’s role as a vector for CL, only 13.9% knew the criteria for distinguishing sand flies from other flies [55]. Overall, the findings showed that the rural people in Kutaber district in the study provinces had little understanding of CL and its sand fly vector, as well as a negative attitude towards it. Yet, teenagers, the elderly, and adults aged 25–33 exhibited a higher level of knowledge regarding CL. This difference could be explained by the acquisition of more information over time, based on personal infection history, information from friends, and observation of other CL-infected persons.

In this study of the multivariate analysis, male participants knew more about CL than female participants, which could be explained by the fact that the male population is with higher trends of infection with CL in the study area. This result is supported by the finding from north central and northwestern parts of the country [20,27]. Humans live in close proximity to animals in rural Kutaber districts, including the study areas. Main home dwellings are traditionally occupied by animals, especially cows. Some households also have spaces for sheep inside or alongside the human dwellings, providing favorable breeding sites for sand flies in the household, even though the smoke inside homes may threaten the sand flies [9].

In the present preliminary entomological survey, five species of sand flies including *Phlebotomus longipes*, *P. sergenti*, *S. squamipleuris*, S*. bedfordi*, and *S. africana* were identified (Table 5). The sand fly fauna detected in the present study area agrees with earlier studies in different parts of Ethiopia [[56], [57], [58], [59]]. Among *Phlebotomus* spp., *P. longipes* was the dominant spp, constituting 80% of total sand fly captures. This species is the proven vector of *L. aethiopica* in northern and central Ethiopia, and Kenya [8,10,[60], [61], [62], [63], [64]]. The predominance of *P. longipes* in the present collection could be attributed to the greater availability of suitable resting and breeding habitats such as the presence of caves, cliffs, and gorges near the presence of cracks in most residential areas of the community [4,10].

In addition, *P. sergenti* was collected, and this species was recognized as a potential vector of CL similar to *L. tropica* in North Africa, Middle East, Afghanistan, Iran and Transcaucasia and Eastern Mediterranean region [30]. In the Awash valley of Ethiopia, *L. tropica* and *L*. *aethiopica* were isolated and characterized [65]. However, the role played by this species in the epidemiology of the CL in this particular focus remains unclear. Moreover, three species of *Sergentomyia* were collected and identified. The most abundant species was *Sergentomyia bedfordi* (9.5%), followed by *S. africana* (5.7%), and *S. squamiplueris* (2.9%). The sand fly fauna found in this study is generally in consistent with prior reports from other parts of Ethiopia [34,56,57,42,66]. On the whole, relatively small number of collected sand fly samples in this study might be associated with seasonality, duration of the study period, and various bionomics of the sand fly signaling the need to undertake further entomological investigations.

## 5 Conclusions

The retrospective data generally showed a slight decreasing trend during the study period. This finding could have several consequences for disease control. Any control measures had no effect in the study area since the high prevalence of CL cases has remained consistent over time. *P. longipes* was the dominant species in the district. Given the abundance of vectors in the study district, this is critical in the selection and implementation of successful vector control measures. There is also a lack of awareness regarding the cause, transmission, treatment, and prevention of CL in the rural community of Kutaber, northeast Ethiopia, as well as a lack of knowledge about the sand fly as a vector for CL. These findings appear to be a direct result of the less attention given to the disease, and a lack of priority given to control measures. In view of the findings from this study, there is a critical need for health education to raise awareness and clarify misunderstandings about all aspects of CL in endemic areas to minimize its incidence and prevalence.

## Author contribution statement

Abib berhanu: Conceived and designed the experiments; Analyzed and interpreted the data; Contributed reagents, materials, analysis tools or data; Wrote the paper.

Sisay Dugassa; Minwuyelet Maru Temesgen; Abebe Animut; Berhanu Erko; Asrat Hailu: Contributed reagents, materials, analysis tools or data; Wrote the paper.

Araya Gebresilassie: Conceived and designed the experiments; Contributed reagents, materials, analysis tools or data; Wrote the paper.

## Data availability statement

Data included in article/supplementary material/referenced in article.

## Declaration of competing interest

The authors declare that they have no known competing financial interests or personal relationships that could have appeared to influence the work reported in this paper
