# Supplementary material for: Cutaneous leishmaniasis in Kutaber District, Ethiopia: Prevalence, sand fly fauna and community knowledge, attitude and practices
Source: Heliyon. 2023 Jul 14;9(8):e18286. doi: 10.1016/j.heliyon.2023.e18286 (PMC10382297; doi:10.1016/j.heliyon.2023.e18286)
Supplement: Multimedia component 1 [file mmc1.docx]

**QUESTIONNAIRE**

A community-based independent study on the knowledge, attitude, and practice towards cutaneous leishmaniasis among selected rural communities in endemic areas of Kutaber district, North-East Ethiopia.

**Identification No.**

Address (village name): ………………………….

1. **PERSONAL INFORMATION**

Age:

…………… years

Sex: Male Female

Religion: Orthodox Christian

Muslim

Others

Unable to write and read

Education: No formal education

Primary

Pre-secondary

Secondary

Preparatory

Tertiary

No. of family members in the same

household:

Occupation:

2

3-5

>5

Government employee

Private employed Farmer Student

Housewife Not working

Marchant

Marital status: Married Unmarried Divorced
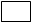
 Widowed

Do you possess a farmland? Yes No

How many years do live in your village? 1-2yrs 3-5yrs >5yrs

Have you travelled out of your village in the last six months? Yes No

**2. KNOWLEDGE ABOUT CUTANEOUS LEISHMANIASIS**

Have you ever seen individuals infected with CL? Yes No I don’t know

What are the signs and symptoms of leishmaniasis? Skin wound

Lesion

Emaciation

Scar

Itching and redness

Skin ulcer

I don’t know

What do you think the mode of transmission of CL? Sandfly bite

By other flies

Auto infection

Bodily contact with patients

I don’t know

In which season is CL more common? Summer Autumn

Winter Spring

I don’t know

Which treatment options do you use in your village? Cauterizing

Chemotherapy

Herbal medicine

Religious solution

I don’t know

How can you prevent CL in your village? Eradicating vector

Insecticide treated nets

Treating patients

Traditional medication

Isolating patients

Improving awareness

I don’t know

Overall knowledge about CL: Good Poor

**3. KNOWLEDGE ABOUT CL VECTOR**

Can you identify sand flies from other

flies and mosquitoes Yes No

if yes? Please tell me how ……………………, …………………………., ……………….

How the CL can be transmitted? Sandfly bite

By other flies

Auto infection

Bodily contact with patients

I don’t know

In which places do sand flies can be commonly found? Everywhere

cracks in basalt cliffs

fissures

holes in walls, barns, caves used by hyraxes

rodent burrows

termite nests

soil cracks

tree trunks

I don’t know

During what hours of the day sand flies can bite individuals?

Morning Night Day time Any time

From dusk to sunrise Don’t know

-How the sand flies can be controlled? Use of insecticides on animal shelter

space-spraying

insecticide-treated nets

personal hygiene

personal protection through application of repellents

I don’t know

Overall knowledge about sand fly vector: Good Poor

**4. ATTITUDE TOWARDS CUTANEOUS LEISHMANIASIS**

Is CL a problem in your area? Yes No I don't know

Is CL more dangerous than Malaria? Yes No I don’t know

Is CL treatable? Yes No I don’t know

Is CL curable? Yes No I don’t know

What do you think the outcome of CL if not treated early?

Death Disability Self cure Others

Is CL preventable? Yes No I don’t know

How is leishmaniasis transmitted? Through sandﬂy bite

Bodily contact with patients

By other ﬂies

I don’t know

From where you came to know about CL?

Family Friends School Media History of infection

Don’t remember Other: ………………………….…………….

Have you ever participated in CL control activities? Yes No I don’t know

Overall attitude: Positive Negative

**-Thank you for your kind cooperation-**

**For any inquiry: use the following address**

**Abib Berhanu**

**E-mail:** [**behoneybal@yahoo.com/abib.abera@gmail.com**](mailto:behoneybal@yahoo.com/abib.abera@gmail.com)

**Tel: +251911155352/+251922842895**
