# Supplementary material for: Cutaneous leishmaniasis in Kutaber District, Ethiopia: Prevalence, sand fly fauna and community knowledge, attitude and practices
Source: Heliyon. 2023 Jul 14;9(8):e18286. doi: 10.1016/j.heliyon.2023.e18286 (PMC10382297; doi:10.1016/j.heliyon.2023.e18286)
Supplement: Multimedia component 2 [file mmc2.docx]

Table 1: The prevalence of CL among patients who visited the Boru Meda Hospital (BMH) Dermatology Department, tallied and counted (from 2015 to March 2021).

| **Year** | **Examined** | | | **Positive cases** | | |
| --- | --- | --- | --- | --- | --- | --- |
|  | Male, n (%) | Female, n (%) | Total | Male, n (%) | Female, n (%) | Total |
| 2015 | 3344 (49.4) | 3432 (50.6) | 6776 | 380 (5.6) | 320 (4.7) | 700 (10.3) |
| 2016 | 4010 (50.7) | 3901 (49.3) | 7911 | 790 (10.0) | 928 (11.7) | 1718 (21.7) |
| 2017 | 5473 (51.3) | 5203 (48.7) | 10676 | 840 (7.9) | 943 (8.8) | 1783 (16.7) |
| 2018 | 6200 (49.1) | 6432 (50.9) | 12632 | 920 (7.3) | 980 (7.7) | 1900 (15.0) |
| 2019 | 7768 (48.2) | 8332 (51.8) | 16100 | 1180 (7.3) | 1090 (6.8) | 2270 (14.1) |
| 2020** | 6106 (46.6) | 7002 (53.4) | 13108 | 590 (4.5) | 545 (4.2) | 1135 (8.7) |
| 2021* | 2059 (50.0) | 2063 (50.0) | 4122 | 264 (6.4) | 232 (5.6) | 496 (12.0) |
| Total | 34960 (49.0) | 36365 (51.0) | 71325 | 4964 (7.0) | 5038 (7.1) | 10002 |

CL, cutaneous leishmaniasis; n, number; %, percent; ** covid year data; *, data was collected only up to March 2021.

Table 2: The retrospective data of CL among the age groups from Boru Meda Hospital, tallied and counted (until March 2021).

| Age groups | Positive case | Negative case | Total |
| --- | --- | --- | --- |
| < 1 yr. | 336 | 3839 | 4175 |
| 1-4 yrs. | 630 | 8679 | 9309 |
| 5-14 yrs. | 1666 | 12424 | 14090 |
| 15-29 yrs. | 4579 | 12760 | 17339 |
| 30-64 yrs. | 2332 | 17104 | 19436 |
| >= 65 yrs. | 459 | 6517 | 6976 |
| Total | 10002 | 61323 | 71325 |
